# Computational Tools for Handling Molecular Clusters: Configurational Sampling, Storage, Analysis, and Machine Learning

**DOI:** 10.1021/acsomega.3c07412

**Published:** 2023-11-14

**Authors:** Jakub Kubečka, Vitus Besel, Ivo Neefjes, Yosef Knattrup, Theo Kurtén, Hanna Vehkamäki, Jonas Elm

**Affiliations:** † Aarhus University, Department of Chemistry, Langelandsgade 140, Aarhus 8000, Denmark; ‡ University of Helsinki, Institute for Atmospheric and Earth System Research/Physics, Faculty of Science, P.O. Box 64, Helsinki 00140, Finland; § University of Helsinki, Institute for Atmospheric and Earth System Research/Chemistry, Faculty of Science, P.O. Box 64, Helsinki 00140, Finland

## Abstract

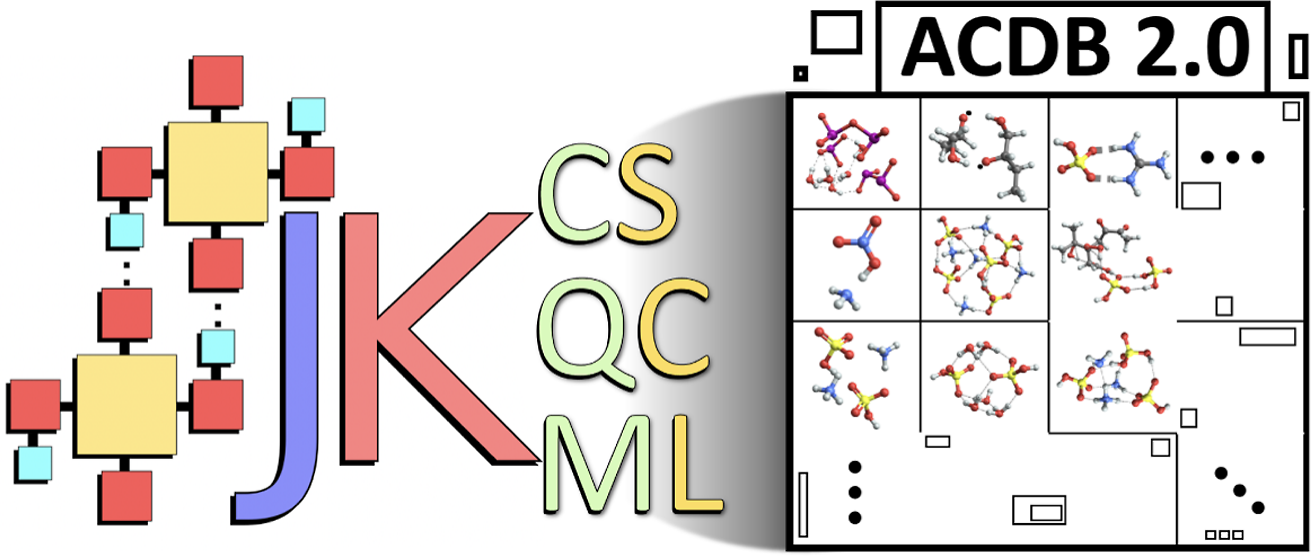

Computational modeling of atmospheric molecular clusters requires a comprehensive understanding of their complex configurational spaces, interaction patterns, stabilities against fragmentation, and even dynamic behaviors. To address these needs, we introduce the Jammy Key framework, a collection of automated scripts that facilitate and streamline molecular cluster modeling workflows. Jammy Key handles file manipulations between varieties of integrated third-party programs. The framework is divided into three main functionalities: (1) Jammy Key for configurational sampling (JKCS) to perform systematic configurational sampling of molecular clusters, (2) Jammy Key for quantum chemistry (JKQC) to analyze commonly used quantum chemistry output files and facilitate database construction, handling, and analysis, and (3) Jammy Key for machine learning (JKML) to manage machine learning methods in optimizing molecular cluster modeling. This automation and machine learning utilization significantly reduces manual labor, greatly speeds up the search for molecular cluster configurations, and thus increases the number of systems that can be studied. Following the example of the Atmospheric Cluster Database (ACDB) of Elm (ACS Omega, 4, 10965–10984, 2019), the molecular clusters modeled in our group using the Jammy Key framework have been stored in an improved online GitHub repository named ACDB 2.0. In this work, we present the Jammy Key package alongside its assorted applications, which underline its versatility. Using several illustrative examples, we discuss how to choose appropriate combinations of methodologies for treating particular cluster types, including reactive, multicomponent, charged, or radical clusters, as well as clusters containing flexible or multiconformer monomers or heavy atoms. Finally, we present a detailed example of using the tools for atmospheric acid–base clusters.

## 1 Introduction

Studying the formation and stability of molecular clusters has been of interest in many scientific domains, such as atmospheric chemistry,^1−6^ biology,^7,8^ astronomy,^9,10^ and material science.^11,12^ A particularly illustrative example is the study of molecular clusters formed in the atmosphere via gas-to-particle conversion, which is the first step in the formation of secondary atmospheric aerosol particles.^13−20^ Once formed, these aerosols have a significant impact on climate and air quality and thus also on human health.^21−23^

Molecular clusters can be experimentally detected,^24−26^ but cluster observations are generally complicated due to a variety of issues such as the typically very low concentrations (often below the detection limit of instruments), the changes a cluster undergoes inside the instruments,^27,28^ and clusters being too small to detect. Many key properties strongly depend on the cluster configuration, which is a priori unknown and is seldom directly measurable. Hence, computational chemistry is an important additional tool to study clusters. Typically, quantum chemical calculations are required to obtain accurate cluster geometries, energies, charge distribution, and so on, which are used to understand the cluster’s stability against fragmentation/evaporation or its potential growth into larger clusters/particles. Unfortunately, quantum chemical (QC) calculations are computationally expensive and the cost steeply grows with cluster size. Configurational sampling, the process of searching for the most relevant cluster configurations, is another bottleneck in molecular cluster studies, as the configurational space quickly grows in complexity with the size of the cluster and the flexibility of its monomers. Several programs exist to explore the vast configurational space of clusters (e.g., ABCluster,^29,30^ OGOLEM,^31^ and CREST^32^). Additionally, many programs for performing different types of QC calculations are available (e.g., Gaussian,^33^ ORCA,^34,35^ XTB,^36−39^ and Turbomole^40^). Configurations can be manually passed from one program to another, but this is cumbersome and error-prone. Therefore, we present the Jammy Key for configurational sampling (JKCS) script that interfaces with the most commonly used third-party programs in the molecular cluster community, manages job submissions to computer clusters via the SLURM job scheduler, and handles the manipulation of the large number of files produced during the process. It further offers tools for data storage and analysis such as filtering, extraction of cluster properties from the output of the QC programs, and QC postcorrection calculations.

Machine learning (ML) methodologies have proven to be highly advantageous due to their capacity to replace time-intensive QC calculations. Several recent studies have harnessed ML techniques to investigate molecular clusters.^41−48^ To assist in the creation of ML models for molecular cluster studies, we introduce Jammy Key for machine learning (JKML). JKML streamlines the training of ML models and their various applications such as predictions of energy/forces and even the creation of ML-based calculators. These calculators can effectively replace QC programs, enabling swift geometry optimization and molecular dynamics simulations, closely replicating the potential energy surface that was used to train the ML calculator. To train ML models, a considerable database of training data is necessary. We have, therefore, upgraded the Atmospheric Cluster Database (ACDB), originally introduced by Elm.,^49^ to ACDB 2.0. In the new ACDB 2.0, cluster properties are stored in a single compressed file, which is easily manipulated by JKQC to use within JKML. The combination of JKCS, JKQC, JKML, and ACDB 2.0 provides the necessary tools to efficiently model a large variety of molecular clusters.

## 2 Methodology

We summarize the working principles of the three main functionalities of the Jammy Key framework: Jammy Key is used for configurational sampling (JKCS), quantum chemistry (JKQC), and machine learning (JKML). Additionally, we discuss how the Jammy Key framework is used to create an improved database for atmospheric clusters (ACDB 2.0).

### 2.1 Configurational Sampling

**Figure 1:**
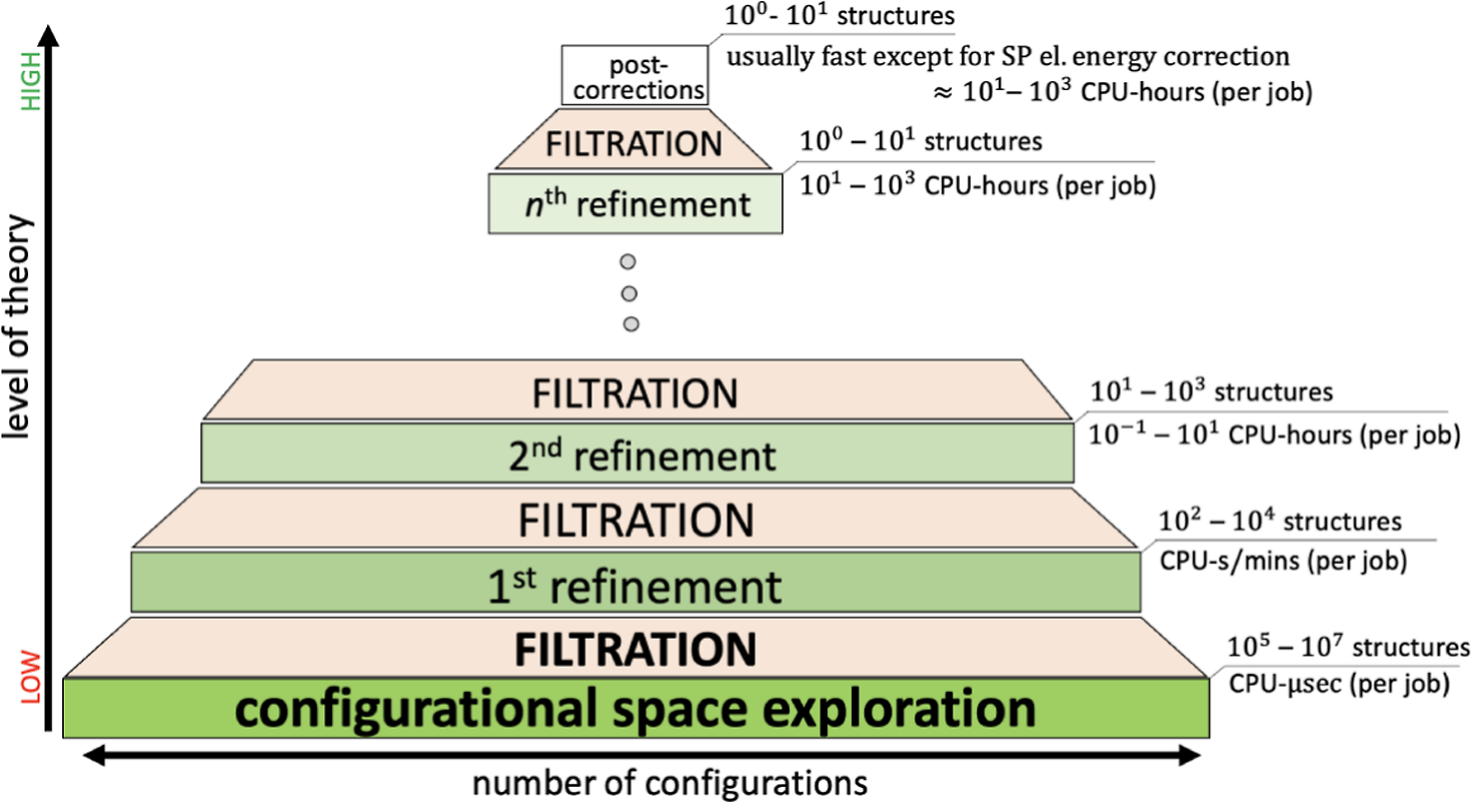
Schematic diagram of the bottom-up approach for conformational sampling.^50^ This figure has been adapted from the dissertation of Kubečka.^51^

Direct examination of complex cluster configurational spaces at a desired high level of theory costs an immense amount of computational resources. Hence, the most common general strategy for configurational sampling (CS) is the so-called bottom-up approach, as illustrated in (Figure 1).^50^ This approach is sometimes also referred to as the “building up” principle, but should be distinguished from strategies where the properties of the *N*-cluster need to be known to calculate the properties of the (*N* + 1)-cluster (an example of such a method is that introduced by Kildgaard et al.^52,53^ to study hydration of dry molecular clusters). The bottom-up approach explores the configurational space using fast but less accurate methods, where only promising candidates are carried through several steps of reoptimization at higher levels of theory and filtering up to the desired level of theory. This methodology determines the workflow of the JKCS scripts.

#### 2.1.1 System Setup

**Figure 2:**
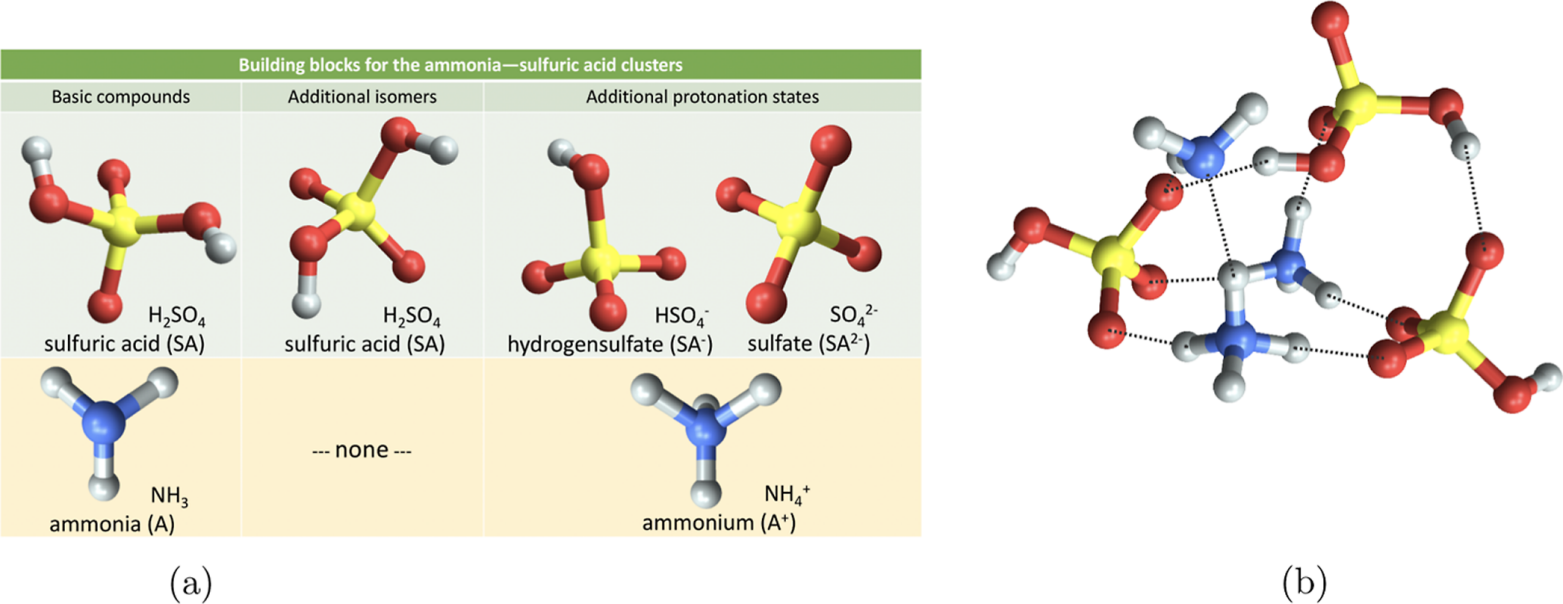
Illustration of all possible building block structures when constructing any ammonia—sulfuric acid clusters. Color coding: H (white), S (yellow), O (red), and N (blue).

System setup involves preparation of the input file (JKCS0_copy copies the default input file into the working directory), where the cluster composition, charge, and spin multiplicity are defined. Typically, individual molecules, denoted as monomers, are used as the initial building blocks. Depending on the type of exploration, either flexible or rigid monomers are used. In the former case, only one conformer for each species needs to be supplied. In the latter case, including the lowest energy conformer of each monomer is a good starting point but adding other conformers and/or assorted protonation states improves the exploration.^54^Figure 2 depicts such a combination of rigid monomers to construct the (H_2_SO_4_)_3_(NH_3_)_3_ cluster. JKCS contains these building blocks for the most common atmospheric cluster forming acid and base molecules and takes care of combining all feasible monomer conformations and protonation states while adhering to stipulated criteria regarding cluster size (number of molecules) and charge. Further, JKCS1_prepare creates one folder for the CS of each cluster type.

#### 2.1.2 Exploration

Configurational space exploration is generally performed at a low level of theory, that is, molecular mechanics or semiempirical methods (e.g., GFN-xTB^36−39^). JKCS (JKCS2_explore) currently communicates with two commonly used configuration space exploration programs: ABCluster^29,30^ and CREST.^32^ ABCluster employs the genetic artificial bee colony algorithm^55^ and can be used with either rigid or flexible monomers. Rigid monomer exploration, often done at the molecular mechanics level, allows for inexpensive and fast exploration of large configurational spaces. The monomer rigidity guarantees that unwanted reactions do not take place (at least at the exploration stage) but also prevents proton transfers or conformational changes essential for cluster stability. Hence, introducing a combination of various building blocks in the system setup allows for a more thorough exploration. Starting from flexible monomers, ABCluster offers a slower exploration combining both cluster configuration and monomer conformation, and this search is typically performed using the GFN-xTB method. In CREST, the configurational space is explored through metadynamics simulations, again often using the GFN-xTB method. The choice of method is highly dependent on the studied cluster. At the end of the exploration, the energetically lowest-lying minimum structures are saved for further refinement.

#### 2.1.3 Refinement

The JKCS3_run script allows communication with QC programs to refine the cluster geometries and energies. Since the number of trial structures obtained from the exploration step can be enormous, the first optimization step should ideally be performed using a computationally affordable method. One of the extended tight-binding (xTB) semiempirical methods implemented in the XTB program^36−39^ is a robust choice for many systems, though caution is needed for reactive and radical systems. The PM6^56^ and PM7^57^ methods offer similar functionality. Subsequent single-point energy refinement or geometry reoptimization can be performed using composite electronic methods such as B97-3c^58^ and r^2^SCAN-3c.^59^ For instance, Engsvang et al.^60,61^ and Wu et al.^62^ showed the applicability of these methods to large (up to 30 molecules) sulfuric acid and ammonia clusters. Nevertheless, a higher level of theory is often required to obtain accurate cluster geometries. For instance, density functional theory (DFT) methods, such as ωB97X-D/6-31++G(d,p),^63^ have been successfully used to describe inorganic molecular clusters.^6^ JKCS communicates with third-party programs such as XTB,^36−39^ ORCA,^34,35^ and Gaussian^33^ and manages the calculation communication. The Jammy Key framework allows us to perform jobs on the user’s local/login computer or on a computer cluster via SLURM job scheduler submission while offering various ways of job distributions, serializations, and parallelizations.

#### 2.1.4 Data Filtering

Filtering is needed to reduce the set of structures passed on by each step as the computational cost per structure can increase by many orders of magnitude from one step to the next. Filtering should, at a minimum, remove redundant (identical or nearly identical configurations) and energetically high-lying configurations, as well as obviously unphysical structures (e.g., clusters that have fragmented or undergone unwanted reactions). Caution is advised when applying energetic filtering using specific cutoff values. For instance, low-level QC methods may predict the relative energies incorrectly, and a good filtering algorithm should take this into account. Therefore, the choice of appropriate filtering criteria is as important as the choice of methods at different levels of the bottom-up approach.

Here, the Jammy Key framework uses the JKQC script (as further described in the next chapter). JKQC allows users to filter structures based on both energetic and structural criteria, for example, the radius of gyration can be used to filter out molecular clusters that have fragmented during optimization. One can, for instance, filter out all structures that have a radius of gyration greater than 10 Å, and energy of *x* kcal/mol higher than the lowest energy found. Appropriate values for *x* require a benchmark examination, as they depend on the type of system, the cluster size, as well as the level of theory used at the step preceding the filtering. For example, Kubečka et al.^54^ used a threshold of 5*M* kcal/mol for filtering after the XTB optimization step in their study on very strongly bound sulfuric acid–guanidine clusters, where *M* is the number of molecules in the cluster. However, lower values (e.g., 2.5*M* kcal/mol) may be appropriate for more weakly bound clusters. The energy threshold should use a high enough cutoff to account for energy reordering due to differences between the different QC methods and possible reordering due to postcorrections.

If two clusters have identical/similar properties, then only one of the configurations should be passed to the next step. This duplicate check can be done by comparing the chosen set of cluster properties such as the radius of gyration *R*_g_, cluster energy *E*, or dipole moment μ. A slower but more accurate option is to compare root-mean-squared displacement (RMSD) between two identically oriented structures, which utilizes a modified version of the ArbAlign program.^64^ Finally, we provide the *selection* method introduced by Kubečka et al.,^54^ which selects a subset of the most distinct configurations from a large data set. This representative subset of configurations might result in not finding exactly the global minimum at the desired final level of theory; however, the best of the selected structures will be close to the real global minimum structure. Such a method is especially useful for large clusters, for which many low-lying energy minima are thermodynamically populated due to small energy gaps between them.

#### 2.1.5 Postcorrections

Another category of data analysis performed via the JKQC script is the post-QC corrections. These can be separated into several subgroups:

- Thermal corrections, performed using the same method as used for geometry optimization, involve mainly vibrational frequency calculations and their contribution to the partition function along with the translational and rotation partition function calculation. Here, JKQC offers to check or correct for:
  Imaginary vibrational frequencies as they indicate that the geometry was not optimized to a minimum. In that case, we attempt several geometry reoptimizations and, if unsuccessful, discard the structure.
  Low-vibrational frequencies, as showed by Grimme,^65^ are caused by treating the vibrations as harmonic, and can lead to unrealistically low free energies. The quasi-harmonic approximation (QHA) corrects the rigid-rotor harmonic oscillator (RRHO) by replacing low-vibrational contributions to entropy with internal rotational modes.
  Vibrational anharmonicity is often just corrected by a scaling factor typical for each QC method applied to all vibrations.^66^ We note that actual anharmonic vibrational frequencies can be calculated with many QC programs, but this is accompanied by numerical stability issues and is rarely feasible or cost-effective for molecular clusters.
  Temperature in QC programs is by default set to 298.15 K (adjustable). JKQC swiftly recalculates thermal corrections at any temperature.
  The rotational symmetry number is often not correctly recognized by QC programs due to computer precision or too high symmetry quantifier thresholds. Although often less important for clusters, the thermodynamic properties of monomers should be either calculated at the correct symmetry or corrected for the rotational symmetry error after QC programs. Here, we recommend the SYMMOL^67^ program, which suggests the maximum symmetry group at a given tolerance.
- Electronic energy may need to be corrected by a single-point electronic energy calculation. This is not needed if a high-level method with a large enough basis set is used. Although the DFT method of our choice, ωB97X-D/6-31++G(d,p)^63^, provides accurate geometries and thermal contributions, the electronic energy must be corrected at a higher level of theory. For instance, cost-effective domain-based local pair natural orbital (DLPNO) variants of coupled cluster methods such as DLPNO-CCSD(T_0_)/aug-cc-pVTZ^68−70^ with NormalPNO criteria can be utilized to calculate Gibbs free energies as^52,66^
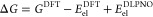
1
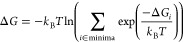
2

- Several energetically close but distinct configuration minima may be populated. In such a case, the lowest free energy minimum does not always sufficiently represent the average cluster structure or its properties. The entropy contribution of all energetically low-lying minima can be accounted for using the Boltzmann distribution, resulting in an average Gibbs free energy.^71^ Nevertheless, here we assume that clusters have a crystal-like behavior and presume that the transition between different minima has a minor effect on the free energies. The problem of clusters with liquid-like behavior, populating many different low-lying free energy minima, should be addressed in future studies.

### 2.2 QC Data Handling, Storing, and Analysis

We present the Jammy Key for Quantum Chemistry (JKQC) Python script designed to store essential molecular cluster information into a Pandas^72,73^ data frame. Structure parsing is accomplished with ASE,^74^ while pertinent molecular properties are directly extracted from QC output files. This approach replaces numerous QC output files, potentially consuming multiple gigabytes of memory, with a single compact file of a few megabytes. Additionally, all the data filtering and postcorrections elaborated in the previous section can be easily performed using JKQC. JKQC is also automated to create input for other programs such as the Ion Mobility Software Suite (IMoS^75^) used to calculate collision cross sections and ion mobilities and the Atmospheric Cluster Dynamics Code (ACDC^76,77^) used to calculate cluster/particle formation rates based on cluster population dynamics. This automation reduces the human errors that accompany the manual construction of these files.

**Figure 3:**
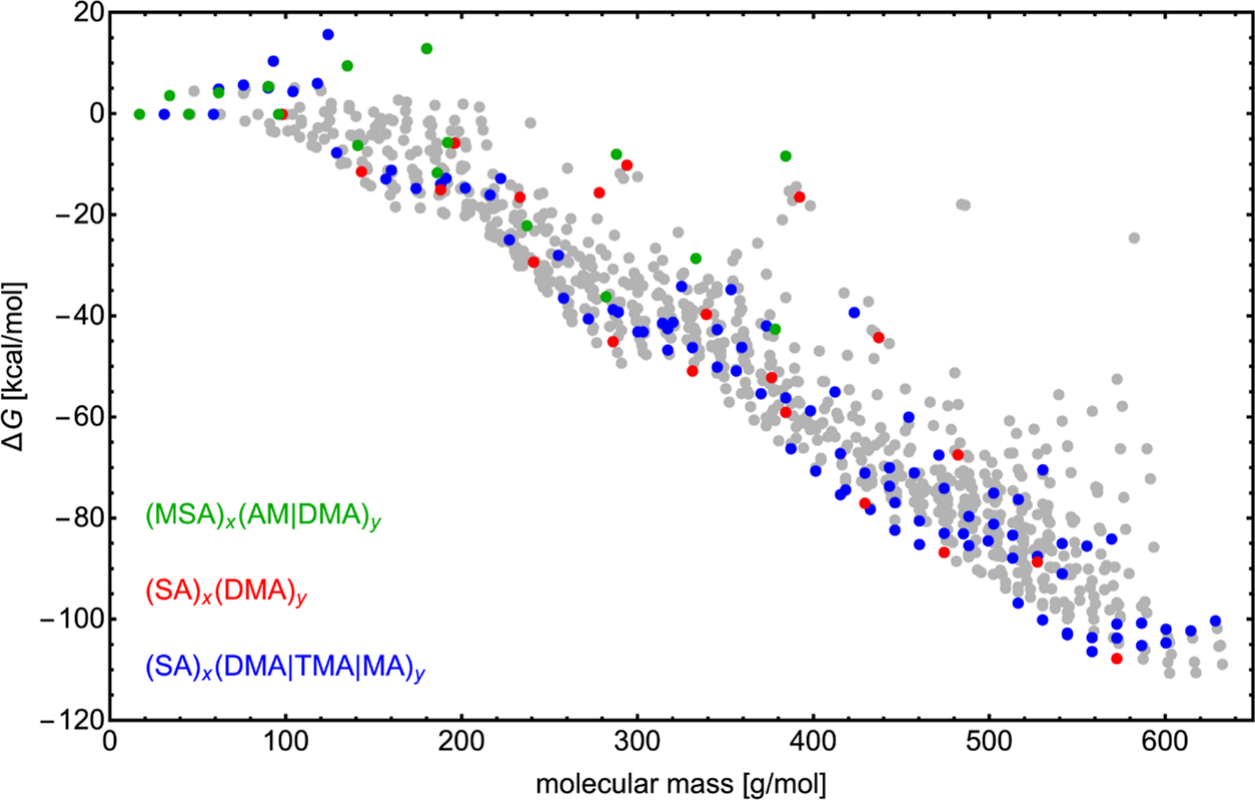
Lowest binding free energies of all cluster types stored in the Atmospheric Clusters Database 2.0 at DLPNO-CCSD(T_0_)/aug-cc-pVTZ//ωB97X-D/6-31++G(d,p) level of theory. Three cluster types are highlighted with different colors: where MSA = methanesulfonic acid, SA = sulfuric acid, AM = ammonia, DMA = dimethylamine, MA, methylamine, and TMA = trimethylamine.

Elm^49^ recently established the ACDB. This database contains clusters composed of molecules responsible for atmospheric new particle formation (NPF): acids (e.g., sulfuric and nitric), bases (e.g., ammonia and dimethylamine), water, and, as of yet, only a few organic molecules. We gathered the ACDB database alongside additional cluster structures and properties from over 30 publications into a new database, ACDB 2.0. Rather than the SDF files of the original ACDB, ACDB 2.0 is constructed as compressed pickle files that contain a large number of cluster properties and are easily read and manipulated by JKQC. We will continuously update the database with the most recently published data. The database currently encompasses more than 1 million entries, spanning various levels of theory. For instance, ∼100k single-point energies are now available at the ωB97X-D/6-31++G(d,p)^63^ level of theory. ACDB 2.0 offers a comparison of different properties between a large set of atmospherically relevant clusters. Figure 3, for instance, depicts the lowest binding free energies of ∼1.5k different cluster types at the DLPNO-CCSD(T_0_)/aug-cc-pVTZ//ωB97X-D/6-31++G(d,p) level of theory with NormalPNO criteria and with QHA and anharmonicity corrections applied. Highlighting three specific cluster types, the figure underscores the potential stability of sulfuric acid–base molecular clusters, illustrating their central contribution to atmospheric NPF. ACDB 2.0 furthermore acts as a foundation for ML applications.

### 2.3 Machine Learning

#### 2.3.1 ML Model

The recent explosion of ML utilization in quantum chemistry has shown that ML potentials can mimic potential energy surfaces within the chemical accuracy of QC methods. There are several ML techniques for regression tasks, such as artificial neural networks (NN),^78^ Gaussian process regression (GPR),^79^ and kernel ridge regression (KRR),^80^ each with their own strengths and weaknesses.^81^ The first task in creating an ML model is choosing the molecular representation for the studied system. Such a representation should be invariant to transformations that do not change the particular property (translation, rotation, mirroring, or nuclear permutation). It should uniquely describe the system and be continuous and, ideally, differentiable.^82^ Commonly used representations for molecular systems are the Coulomb Matrix (CM),^83^ Bag of Bonds (BoB),^84^ Many-Body Tensor Representation (MBTR),^85^ Smooth Overlap of Atomic Positions (SOAP),^86^ FCHL18/19,^87,88^ and those integrated in NN architectures such as SchNet^89^ and PaiNN.^90^ Several ML studies have already been conducted for atmospherically relevant molecular systems. Jääskeläinen showed that ML approaches are useful to improve cluster structure selection and sampling in general.^48^ NNs have been used to model large sulfuric acid–dimethylamine clusters^47^ and the NN potential ANI-2x^91^ has been benchmarked for small dimer clusters.^92^ KRR/GPR has been used to predict cluster binding energies,^44−46,61^ saturation vapor pressures of organic molecules,^41,42^ and chemical potentials of organic molecules in atmospherically relevant solutions.^43^

Our ML-oriented subpackage, JKML, offers an interface between the JKQC-constructed database files (e.g., those stored in ACDB 2.0) and two ML programs, quantum machine learning (QML^93^) and SchNetPack.^94,95^ In the procedure, XYZ coordinates are extracted and together with the property of interest (e.g., electronic energy, forces, or mobility) are stored in a database. Subsequently, JKML uses QML or SchNetPack to perform the training, validation, and testing of the predicted property or its difference from a reference state. In the case of energies, these can be atomization energies for molecules, or binding energies for molecular clusters
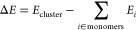
3

In our previous work,^44,46^ we showed that utilizing Δ-ML,^96^ that is, predicting the difference in binding energy between a low and high QC method, increases the accuracy of the model compared to direct-ML. For instance, the difference in electronic binding energy is calculated as
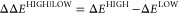
4

KRR can potentially achieve higher accuracy than NN for small databases. On the other hand, NN is accurate and fast for large training databases. Since KRR becomes computationally demanding with increasing database size, training an ML model on energy gradient/forces, that is, 3*N*-times more variables, seems more suitable for NN. However, several KRR-based methods suitable for GPU/TPU also exist (e.g., QML-lightning^97^ and sGDML^98^). For now, JKML allows for the training of the aforementioned NN-based potential utilizing the forces extracted from QC via JKQC. The trained model can be used for geometry optimizations and fast MD simulations. However, for accurate and fast modeling, a training database must be constructed for the system at hand. Additionally, the greater the number of atom types in the studied system, the greater the required training database.

#### 2.3.2 Categorization Trick

JKML allows multinode parallelization for kernel construction within the KRR calculations. However, even with this parallelization, it becomes computationally demanding to train on more than 100,000 data points. For each test structure, we offer training data set reduction based on structure similarity, thus removing the need for training on redundant or unnecessary structures. Here, we utilize the MBTR^85^ representation, as implemented in the DScribe^99^ library, to calculate the distribution of atom-specific bonds and bond angles ρ_MBTR_ of each structure *x*_*i*_. Furthermore, we define the similarity between two structures by calculating the overlap of the two distributions at given bond lengths (*r* ∈ ⟨ 0.7, 2⟩ Å) and eventually also bond angles (α ∈ ⟨ 0, 2π⟩ rad)
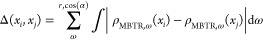
5

The MBTR representation is a discretized (we use a 100-unit grid for bonds and a five-unit grid for angles) function corresponding to a sum of Gaussian functions with a small deviation of σ = 10^–9^, an exponential weighting function of 0.5, and a minimum threshold of 3 × 10^–3^. Gaussian functions are situated around each bond length and/or bond angle for specific atoms. Thus, a value of Δ(*x*_*i*_, *x*_*j*_) = 0 indicates that the structures are identical, and low values determine high similarity. Consequently, one can speed up the ML modeling of a target configuration by training only on a small data set.

## 3 Application and Discussion

### 3.1 Configurational Sampling Obstacles

**Figure 4:**
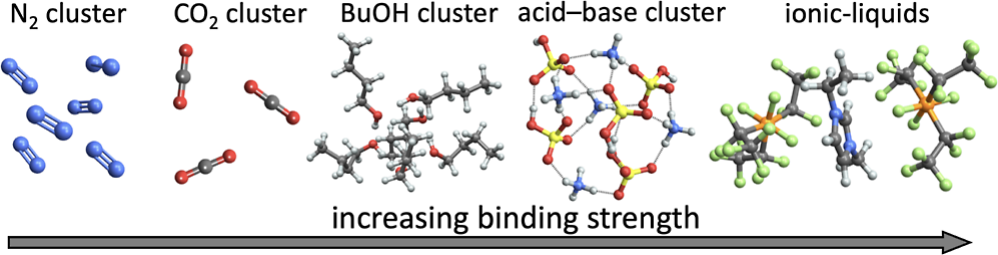
Several examples of molecular clusters sampled by JKCS sorted with respect to their binding energy. Color coding: H (white), O (red), C (gray), S (yellow), N (blue), P (orange), F (lime).

Cluster formation inevitably involves both enthalpy (Δ*H*) and entropy (Δ*S*) changes, and hence the clustering free energy (e.g., Δ*G* = Δ*H* – *T*Δ*S*) increases with increasing temperature (*T*). Weakly bonded clusters thus typically require a low temperature and/or high vapor concentrations to form in the gas phase, while strongly bonded clusters may also be formed at room temperature and trace concentrations of the vapors. Figure 4 illustrates several cluster systems ordered by their binding strength. Examples include noble gas atomic clusters (e.g., helium and argon) and molecular clusters like carbon dioxide (CO_2_), which are stabilized by weak London dispersion interactions. Other clusters composed of methanol (CH_3_OH), butanol (C_4_H_7_OH), or water (H_2_O) are bound relatively more strongly, primarily via hydrogen bonds. On the opposite end, acid–base, NaCl salt, and ionic-liquid clusters are bonded by robust ion–ion Coulomb interactions.

Neither the binding energy nor the reactivity (e.g., proton transfer) alone is sufficient to determine how straightforward the CS process is for a given clustering system. Over several years, JKCS has been used to study various clusters and their properties.^3,6,44,46,54,62,100−113^ Based on these studies, we present typical examples of molecular cluster properties that complicate CS compared to a reference case of a nonreactive, one-component, crystal-like cluster with a single low-energy configuration, formed from rigid, closed-shell monomers with only one conformer. This reference case can be, for example, a water cluster at a very low temperature, that is, an ice crystal. Clusters fall into one or several of the following categories:

**Multicomponent:**

e.g., (H_2_SO_4_)_*x*_(NH_3_)_*y*_(CH_3_NH_2_)_*z*_((CH_3_)_2_NH)_*u*_(H_2_O)_*w*_.

JKCS can construct clusters with an arbitrary number of components. However, the number of dimensions to study increases with the number of components, especially when various different combinations of {*x*, *y*, *z*, *u*, *w*} need to be examined. There is no general solution to this problem. One could potentially lump some monomers^114^ together based on similarity or use ML methods to accelerate the CS.^46,105^ Another option would be to use a lower level of theory along, e.g., the water (*w*) axis.

**Reactive:**

e.g., (organic)_*x*_(H_2_O)_*y*_(O_3_), (R=O)_1_(R–OO·)_1_, (H_2_O)_*x*_(NH_3_)_*y*_.

Intra- and intermolecular reactions (e.g., oxidation, bond breaking, but also proton transfer) can occur within clusters if available through thermal fluctuations. Sampling potential reactants and products of the relevant reactions separately, utilizing reactive potentials within, for example, CREST,^32^ or performing MD simulations are possible solutions. Transition state conformers could also be searched by fixing the reactive area.

**Acid–base:**

e.g., (H_2_SO_4_)_*x*_(NH_3_)_*y*_, (HNO_3_)_*x*_((CH_3_)_2_NH)_*y*_(H_2_O)_*z*_.

This is a subgroup of reactive clusters that undergo proton transfer within the cluster. Using combinations of conformer/protonation states in rigid monomer exploration enables a thorough CS while accounting for all possible proton transfers. An ABCluster^29,30^ search with rigid monomers could also be followed by re-exploration around energetically low-lying structures within the reactive potential via CREST.^32^

**Multiconformer/flexible monomers:**

e.g., (H_2_SO_4_)_*x*_(C_2_H_4_(NH_2_)_2_)_*y*_, (C_4_H_9_OH)_*x*_.

Some molecules can have a large number of conformers. Including all or as many conformers as possible in a rigid monomer exploration guarantees better exploration. We recommend more conformer combinations, that is, more parallel explorations, with short exploration times rather than one long thorough exploration with one conformer combination. Typically, the ABCluster^29,30^ combined global-configuration and conformation search or CREST^32^ search are suitable for this problem.

**Metastable monomer conformers:**

e.g., (organic)_*x*_(H_2_O)_*y*_, (HIO_3_)_*x*_(HIO_2_)_*y*_.

Monomers within clusters can take on configurations that are not stable minima in the gas phase but can exist and even dominate inside clusters, as they are stabilized by the cluster environment. These metastable monomer conformers should be manually constructed and included in the exploration step. They should not be preoptimized as they would only revert to the gas-phase minimum structure. CREST^32^ might be, in some cases, more suitable for this problem.

**Heavy atom(s):**

e.g., (HIO_3_)_*x*_(H_2_O)_*y*_, K_*x*_[Pb(ligand)_*y*_].

Low-level theory energy evaluations may fail for clusters containing heavy atoms (period five or higher in the periodic table) since they might lack a description of relativistic effects, polarization, or other heavy-element-related phenomena. Heavy-atom-related effects should be accounted for in the CS by at least including pseudopotentials during DFT calculations or scalar relativistic Hamiltonians.

**Liquid-like:**

e.g., (H_2_SO_4_)_*x*_(NH_3_)_*y*_(H_2_O)_*z*_, or (Ar)_*x*_ and (CO_2_)_*x*_ at low temperature.

The description of the thermodynamic properties of these clusters is difficult since we cannot use the superposition approximation of the lowest free energy minima. There is a (cluster-type-dependent) threshold temperature above which the energy barriers separating different local energy-minimum conformations become easy to overcome by thermal fluctuation. MD simulations can likely provide insight into this problem; however, further research on this topic is still required.

**Charged clusters or ionized molecules:**

e.g., Cl^–^(H_2_O)_*x*_, (C_*x*_H_*y*_O_*z*_)·NO_3_^–^, (H_2_SO_4_)_*x*_(NH_3_)_*y*_.

Clusters may be charged or contain ionic monomers even when the clusters or monomers are initially neutral (e.g., due to proton transfer; see acid–base clusters). Charges present in clusters cause inductive effects (electron flows) and charge delocalization over several neighboring molecules. Hence, additional polarization and diffuse basis set functions are needed. Moreover, if we require the charge to be localized on a specific cluster molecule (e.g., due to photoionization or charge transfer), additional QC techniques might be necessary such as constrained DFT and various fragmentation methods.

**Unpaired electrons:**

e.g., [(CH_3_)_3_C–O·]_2_, (R–CO–OO·)_1_(H_2_O)_*x*_.

Radical systems can usually be described only by QC methods (not force fields, FF). The potential energy surface can still be explored using FF to obtain a broad set of initial structures, but FF parameters should be carefully checked to prevent, for example, radical centers from being treated as ions. Nevertheless, exploration at, for example, the XTB level of theory is more suitable. Further, high-level QC methods should be used to describe these open-shell systems (freezing problematic parts might be helpful, especially during initial optimizations). The QC results should also be checked for spin contamination, where relevant.

**Figure 5:**
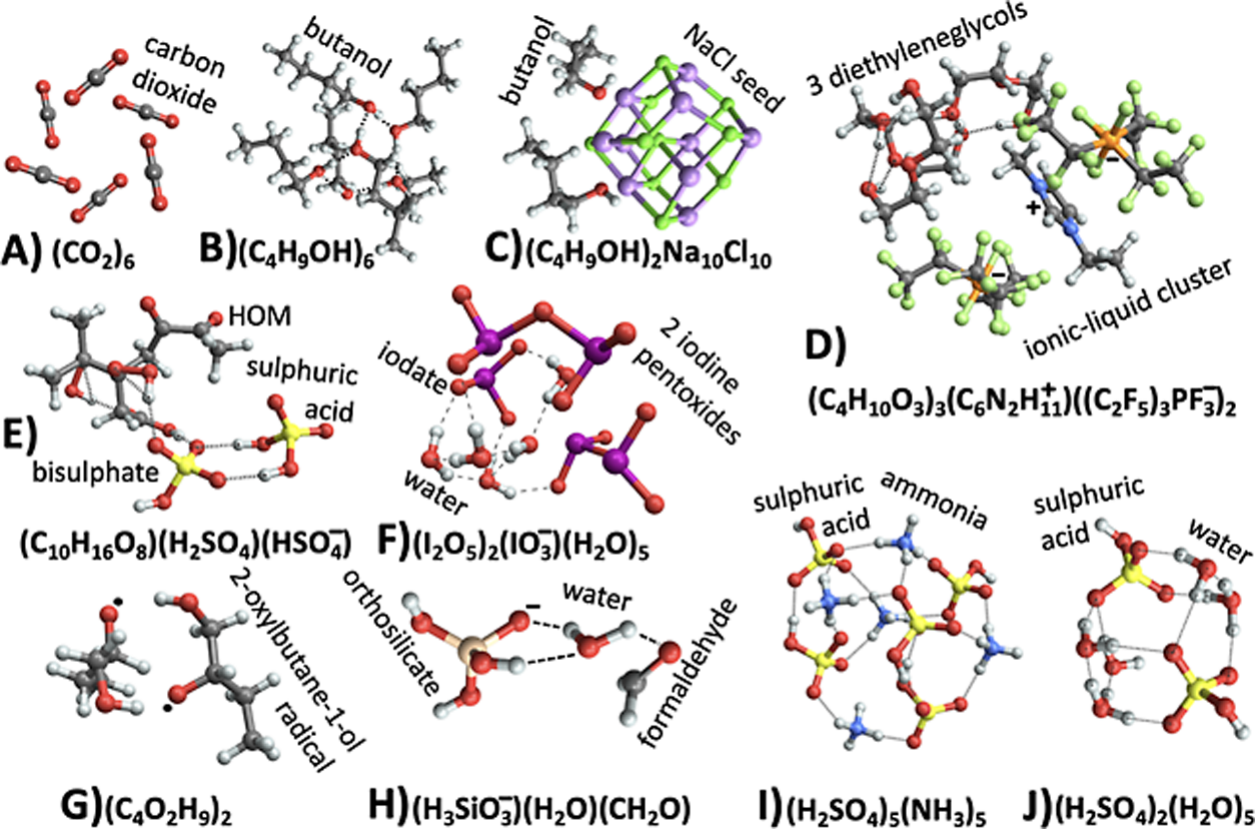
Examples of clusters corresponding to studies are presented in Table 1. Color coding: H (white), S (yellow), O (red), C (gray), I (purple), Na (mauve), Cl (green), N (blue), P (orange), F (lime), Si (cream).

We advise readers to have a look at the articles gathered in Table 1 and Figure 5. These articles used JKCS for various cluster systems, and the CS of these systems is presented along with technical details within the main text or the Supporting Information. However, JKCS has so far found the most applications for CS of atmospheric acid–base molecular clusters, which we focus on in detail in the next section.

**Table 1:** Overview of Several Cluster Studies That Utilized the JKCS Package

**single-component nonreactive clusters**	
**Carbon dioxide clusters at ∼40–90 K**.^106^ Achieving accurate binding energies required challenging QC calculations such as high-level theory (CCSD(T)) along with diverse corrections because lower methods struggled to precisely capture the weak dispersion interactions.	Figure 5A
**Butanol (C_4_H_7_OH) clusters.**(107) Butanol has multiple internal rotations, yielding multiple conformers. Clusters were formed through their random combinations. Modern DFT or even some semiempirical methods provide sufficiently accurate formation thermodynamics for these hydrogen-bonded clusters, eliminating the need for higher-level corrections.	Figure 5B
**multicomponent nonreactive clusters**	
**Butanol and water condensing onto NaCl seed**.^107,108^ We used ABCluster conformation and configurational search. Note that CS of systems with more nonmixing physical phases (e.g., cluster formation on a surface) is currently available within ABCluster after introducing restrictions for phase mixing.^115^	Figure 5C
**Diethylene glycol around ionic-liquid clusters**.^109^ Multiple conformers were introduced at the beginning of CS. With no proton transfer present, interactions were well described by DFT methods with large basis sets.	Figure 5D
**Clusters of sulfuric acid and organic molecules**.^110^ A highly oxygenated organic molecule (HOM)^116^ was only represented by one conformer to speed up the CS. Due to the inherent flexibility of organic molecules, employing multiple representative conformers (e.g., found via the Spartan program^117^) or using CREST^32^ is advisable. Furthermore, assessing the reactivity within such clusters is crucial due to the presence of reactive functional groups, potentially participating in proton transfer.	Figure 5E
**clusters containing heavy atom and metastable monomer**	
**Iodine-containing molecular clusters.**(3,100) Iodic acid has a metastable conformation in clusters. We constructed an extra building block with varied proton orientations. Sequentially, advanced QC methods were used to account for relativistic effects. Conversely, a low level of theory was used for large clusters to obtain approximate configurations for collision cross-section estimation.	Figure 5F
**reactive organic clusters**	
**Dimers of organic alkoxy radicals (formed in peroxy radical self-reactions)**.^111,112^ ABCluster with multiple conformers per monomer was used to obtain trial structures further passed to higher-level theory, which is able to describe radical systems. Nevertheless, using CREST^32^ is advisable for future studies.	Figure 5G
**Accretion reactions on dust particle**.^113^ Dust particles were approximated by a representative molecule. Transition state modeling was performed at a high level of theory to describe the reaction energy barriers.	Figure 5H

### 3.2 JKCS Workflow for Acid–Base Clusters

**Figure 6:**
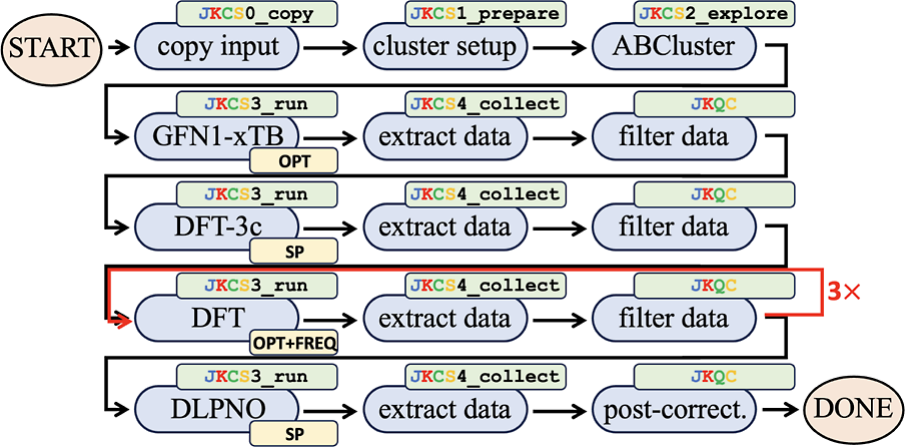
CS workflow used by Knattrup et al.^105^

Under atmospheric conditions, clusters that grow to nanoparticles or aerosols from gas molecules often involve strong acids and bases. After collision, these molecules undergo proton transfer reactions forming strongly bound ion pairs (salt), exemplified by cases such as (H_2_SO_4_)_2_(NH_3_)_2_ → (HSO_4_^–^)_2_(NH_4_^+^)_2_. To accommodate the protonation states of monomers and multiconformer sulfuric acid during CS, the provided building blocks (Figure 2a) are employed. Also, an accurate QC examination of thermodynamic properties necessitates a high level of theory and extra-polarized and diffuse basis functions. These methodologies were used for studies of acid–base clusters such as sulfuric acid–ammonia clusters,^61,62,102^ sulfuric acid–dimethylamine clusters,^62,104^ systems involving trimethylamine oxide,^103^ sulfuric acid–multibase clusters,^46^ and even multiacid–multibase clusters.^105^ For these cluster types, the CS procedure via a multistep funneling approach^6,54^ has been well optimized. For instance, Knattrup et al.^105^ recently used the workflow scheme depicted in Figure 6.

Clearly, this multistep procedure would be cumbersome to do manually. Consequently, its full automation allowed us to explore hundreds of cluster systems. Nevertheless, we recommend that new users perform each command separately and examine its outcome first. Several molecular cluster benchmark studies^45,66,118−121^ can be used to choose a suitable QC method. For instance, we currently use GFN1-xTB implemented in the XTB program,^36−39^ DFT-3c such as B97-3c or r^2^SCAN-3c implemented in ORCA,^34,35^ DFT such as ωB97X-D/6-31++G(d,p)^63^ implemented in the Gaussian^33^ and ORCA programs, and DLPNO such as DLPNO-CCSD(T_0_)/aug-cc-pVTZ^68−70^ with NormalPNO criteria also implemented in the ORCA program. Note that we typically need to restart some DFT calculations, as the minimum was not found. This can be caused by the calculations not converging or by the presence of an imaginary vibrational frequency. The filtering of redundant structures (nonunique, fragmented, reacted, and with too high energy) and all subsequent postcorrections is implemented within JKQC and described in the manual.

Atmospheric molecular clusters often become quickly solvated by a few water molecules due to high air humidity. Therefore, the consideration of hydration becomes essential in the study of atmospheric clusters, which introduce water as an extra component, increasing the CS complexity. Water can also function as both an acid and a base, potentially introducing reactivity through proton transfer reactions. For simplicity, hydration was often omitted in studies involving atmospheric acid–base clusters. However, this introduces an additional source of error in such studies, as the impact of water can enhance cluster formation by up to 2 orders of magnitude.^122^

Rasmussen et al.^101^ demonstrated that for water-containing clusters, the approach of Kildgaard et al.^52,53^ can outperform the JKCS method presented here. Their technique requires knowledge of low-energy structures for “dry” clusters and involves sequentially inserting water between existing bonds or around the cluster, exploring only a fraction of configurational space. While both methods are able to find the global minimum, JKCS demands significantly more computational resources due to its exploration of a larger configurational space. For sizable clusters with distinct predictable bonding patterns, like large hydrate clusters, alternative approaches such as Kildgaard’s method could offer faster CS. We encourage future studies to incorporate water.

### 3.3 ML Potential

**Figure 7:**
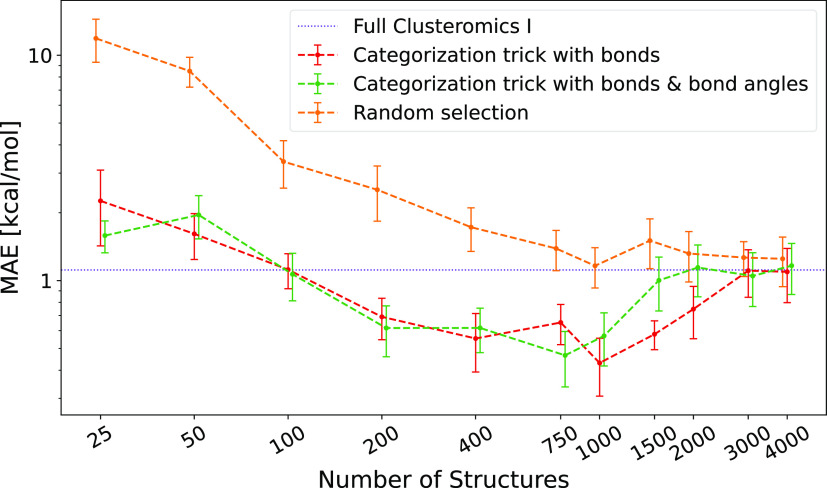
Learning curves, that is, mean absolute error (MAE) functions of the training set size, for the electronic binding energy predictions of 10 (H_2_SO_4_)_3_(base)_3_ clusters from Xie et al.^124^ The learning curves with different training subdatabase selection approaches (red, green, orange) converge to the MAE of training on the full (∼32k) database (black dotted line). Error bars correspond to the standard deviation of the sample mean.

In our recent Clusterome paper,^123^ we presented a large (∼250k), multiacid–multibase, atmospherically relevant, molecular cluster database (available in the ACDB 2.0 repository, see the Supporting Information). Here, we extract only ∼32k structures of the (H_2_SO_4_–SA)_0–2_(bases)_0–2_ clusters (termed Clusteromics I), where bases correspond to ammonia (AM), methylamine (MA), dimethylamine (DMA), trimethylamine (TMA), and ethylenediamine (EDA). We trained the KRR potential with the FCHL19^88^ molecular representation (via JKML) to examine whether Δ-ML r^2^SCAN-3c∥GFN1-xTB can substitute the single-point r^2^SCAN-3c in the JKCS workflow mentioned in the previous section. Hence, we selected 10 random equilibrium (H_2_SO_4_)_3_(base)_3_ clusters from Xie et al.^124^ and predicted their binding electronic energies with the ML model. Figure 7 shows that the learning curve (orange line) rapidly converges to a mean absolute error (MAE) of 1.11 kcal/mol, corresponding to the model trained on the full Clusteromics I (black dotted line). Hence, already 1k structures randomly selected from the Clusteromics I database are enough to train an accurate ML potential and replace the r^2^SCAN-3c step in the ML workflow. The training on 1000 structures with a subsequent test on 10 structures takes ∼5 CPU hours. However, performing r^2^SCAN-3c itself takes only ∼1 CPU hour. Since this QC method is computationally quite fast, using ML does not speed up the process. More useful is, for instance, Δ-ML^ωB97X-D∥GFN1-xTB^ as used in our previous work on the CS of SA-multibase clusters^46^ or even Δ-ML^DLPNO∥ωB97X-D^ as suggested in our recent perspective.^81^ However, as a proof of concept, we will use r^2^SCAN-3c in the next section to show the potential ML speedup by the categorization trick.

#### 3.3.1 Fast ML

JKML offers two types of in-house algorithms to speed up KRR modeling. The first, kernel splitting (*-split* ⟨*int*⟩), employs multinode parallelization of kernel matrix constructions. Hence, the same results are obtained with the same computational resources but in a shorter wall-clock time. Second, the “categorization trick” (*-categorize* ⟨*int*⟩), compares the similarity between a test structure and all training and predicts using an ML model trained only on a subset of similar structures, where the similarity is performed via bond (and bond angle) comparison. This leads to faster predictions with an expected minor decrease in accuracy. With more test structures, a new ML model is trained for each tested structure. Another option would be to combine the training subsets into one reduced subset.

Figure 7 shows the learning curves for the selection via the categorization trick (green and red curves). This smart selection from the full database leads to approximately fivefold lower MAEs compared to a random selection. It can even reach lower values compared to the prediction on the full database, as structures that do not resemble the test structure(s) do not bias the model. When the training set size grows, all learning curves converge to the MAE of the full database. Further, we only use the categorization trick based on bonds, as including angles does not seem to improve the categorization trick.

Finally, to provide statistically accountable proof, we again used the ∼32k (SA)_0–2_(bases)_0–2_ clusters (Clusteromics I) for training and tested it on 5k (SA)_3_(bases)_3_ and 1k (SA)_4_(bases)_4_ clusters from Kubečka et al.^46^Table 2 shows that the MAE of the predicted binding energies using the model trained on the full database is similar to the errors of the categorization trick with 100 and 200 trained structures for each target structure. Here, it is worth noting that the MAE of the ML predictions is less important for CS as we sort and filter configurations based on relative energies. Hence, the RMSD (i.e., span of errors) defines the quality of the ML model. As an example of computational times, the training on the full Clusteromics I took ∼152 CPU days and the prediction of the (SA)_3_(base)_3_ set took an additional 103 CPU days (overall 255 CPU days). The equivalent process using the selection trick with 100 and 200 structures took overall only ∼13 and ∼43 CPU days, respectively. Clearly, selecting only 200 training structures in the (SA)_3_(base)_3_ modeling is fast but we could reach even greater accuracy by using ∼800 structures (based on Figure 7). However, this would lead to ∼16 times slower modeling.

**Table 2:** Test of the Categorization Trick (*-categorize*) Method with 100 and 200 Most Similar Structures Selected from the Clusteromics I and Errors in the Prediction of Binding Energies of the Equilibrium (SA)_3_(base)_3_ and (SA)_4_(base)_4_ Clusters (Where SA Is Sulfuric Acid)

train	test	methods	MAE ± RMSD [kcal/mol]
Clusteromics I	(SA)_3_(base)_3_	**full database**	**0.8±1.0**
		*-categorize* 100	1.4 ± 1.7
		*-categorize* 200	1.2 ± 1.5
	(SA)_4_(base)_4_	**full database**	**3.3±5.2**
		*-categorize* 100	3.6 ± 4.7
		*-categorize* 200	2.6 ± 3.7

**Figure 8:**
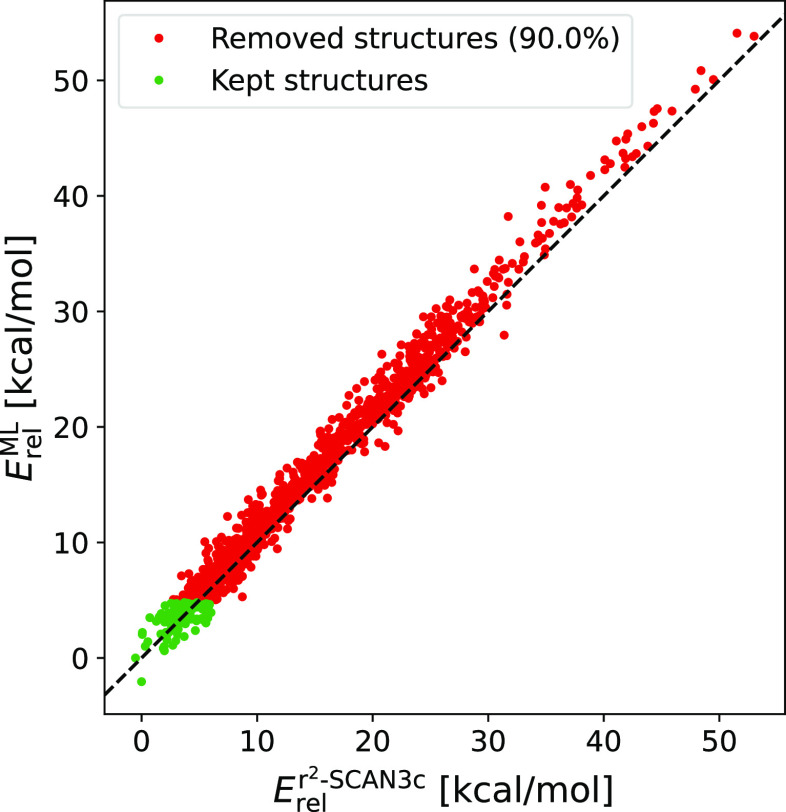
Correlation between Δ-ML and r^2^SCAN-3c relative electronic energies (compared to the lowest conformer) for the equilibrium (SA)_3_(DMA)_3_ clusters (SA = sulfuric acid and DMA = dimethylamine). Here, the Clusteromics I data set was used for training the ML model with the 200-structure categorization trick. Filtering out 90% of redundant structures is highlighted in red color.

To summarize, Δ-ML is able to substitute the r^2^SCAN-3c step in the CS workflow. This could be followed by filtering 10% of the lowest Δ-ML energies to the next step (e.g., DFT optimization). Figure 8 shows the correlation between Δ-ML predicted energies and r^2^SCAN-3c energies for the (SA)_3_(DMA)_3_ cluster, which supports the use of a 200-structure categorization trick. With this single-point energy approach, fewer structures need to be taken to computationally demanding DFT calculation as opposed to filtering straight on the semiempirical energy ordering. With very large diverse databases or training more parameters (e.g., forces), we also recommend using other training data set reduction methods^125^ and/or using JKML coupled with SchNetPack^94,95^ for training a NN potential.

## 4 Conclusions

In this work, we introduced the Jammy Key framework, a collection of scripts designed for systematic CS of molecular clusters as well as their handling, storing, and subsequent analysis. Notably, its core strengths are organized processing and automated file administration. The toolkit interfaces with commonly used third-party software such as ABCluster, CREST, XTB, Gaussian, and ORCA for executing quantum chemistry calculations while retaining adaptability for integration of alternative third-party programs. Its ultimate aim is to identify a representative set of structures corresponding to the lowest free energy minima. We demonstrated the application of the JKCS to various systems, primarily focusing on atmospheric molecular clusters, although the underlying principles are universally applicable.

JKQC is another powerful tool that allows the extraction of coordinates, forces, and other properties from QC programs into a compressed file. JKQC offers further manipulation of the stored data frame including sorting, filtering, specific data printing, applying QC corrections, calculation of binding (and atomization) properties, and producing input files for, for example, the ACDC or the Ion Mobility Software suite (IMoS). Consequently, the analysis of large data sets with JKQC becomes more automated and faster. We used these advantages to upgrade the architecture of the ACDB, conceived by J. Elm. Hence, the new version, ACDB 2.0, contains more cluster descriptions and takes less memory space, and the manipulation of the database is significantly improved. As a result, ACDB 2.0 presently encompasses over 1 million entries of molecular cluster configurations/properties that are suitable for ML applications.

Finally, we introduce the JKML that interfaces with the QML and SchNetPack packages. This interface facilitates the application of ML techniques from the KRR and NN families. We outline the potential integration of ML into the CS procedure and deliberate on their applicability with respect to the training data set size. We believe that incorporating ML techniques holds substantial promise in reducing the computational expenses associated with future investigations of molecular clusters.

## Supporting Information Available

Package availability (PDF)
