# Supplementary material for: Computational Tools for Handling Molecular Clusters: Configurational Sampling, Storage, Analysis, and Machine Learning
Source: ACS Omega. 2023 Nov 14;8(47):45115–28. doi: 10.1021/acsomega.3c07412 (PMC10688175; doi:10.1021/acsomega.3c07412)
Supplement: Supplementary file 1 — ao3c07412_si_001.pdf [file ao3c07412_si_001.pdf]

Supporting Information

for

Computational Tools for Handling Molecular Clusters: Configurational Sampling, Storage, Analysis, and Machine Learning

Jakub Kubečka,[*,†] Vitus Besel,[‡] Ivo Neefjes,[‡] Yosef Knattrup,[†] Theo Kurtén,[¶] Hanna Vehkamäki,[‡] and Jonas Elm[†]

†*Aarhus University, Department of Chemistry, Langelandsgade 140, Aarhus, 8000, Denmark*

‡*University of Helsinki, Institute for Atmospheric and Earth System Research/Physics, Faculty of Science, P.O. Box 64, Helsinki, 00140, Finland*

¶*University of Helsinki, Institute for Atmospheric and Earth System Research/Chemistry, Faculty of Science, P.O. Box 64, Helsinki, 00140, Finland*

E-mail: ja-kub-ecka@chem.au.dk

Phone: +420 724946622

S1 Package availability

The JKCS package comes together with JKQC and JKML and is a free open-source program immediately available at

https://github.com/kubeckaj/JKCS2.1

The package contains Bash and Python codes and is suitable for any GNU/Linux system. Each script contains its own help function and the overall manual with some recommended approaches is available at

https://jkcs.readthedocs.io

The Atmospheric Cluster Database 2.0 is available at

https://github.com/elmjonas/ACDB.git

under the database_v2 subfolder.
